# Survival Strategies in the Aquatic and Terrestrial World: The Impact of Second Messengers on Cyanobacterial Processes

**DOI:** 10.3390/life4040745

**Published:** 2014-11-18

**Authors:** Marco Agostoni, Beronda L. Montgomery

**Affiliations:** 1 Cell and Molecular Biology Graduate Program, 322 Plant Biology Building, Michigan State University, East Lansing, MI 48824, USA; E-Mail: marcoago@msu.edu; 2 Department of Energy Plant Research Laboratory, 106 Plant Biology Building, Michigan State University, East Lansing, MI 48824, USA; 3 Department of Biochemistry and Molecular Biology, Michigan State University, 612 Wilson Road, East Lansing, MI 48823, USA

**Keywords:** cyanobacteria, second messenger, Ca^2+^, (p)ppGpp, cAMP, c-di-GMP, cGMP, nitric oxide, c-di-AMP

## Abstract

Second messengers are intracellular substances regulated by specific external stimuli globally known as first messengers. Cells rely on second messengers to generate rapid responses to environmental changes and the importance of their roles is becoming increasingly realized in cellular signaling research. Cyanobacteria are photooxygenic bacteria that inhabit most of Earth’s environments. The ability of cyanobacteria to survive in ecologically diverse habitats is due to their capacity to adapt and respond to environmental changes. This article reviews known second messenger-controlled physiological processes in cyanobacteria. Second messengers used in these systems include the element calcium (Ca^2+^), nucleotide-based guanosine tetraphosphate or pentaphosphate (ppGpp or pppGpp, represented as (p)ppGpp), cyclic adenosine 3’,5’-monophosphate (cAMP), cyclic dimeric GMP (c-di-GMP), cyclic guanosine 3’,5’-monophosphate (cGMP), and cyclic dimeric AMP (c-di-AMP), and the gaseous nitric oxide (NO). The discussion focuses on processes central to cyanobacteria, such as nitrogen fixation, light perception, photosynthesis-related processes, and gliding motility. In addition, we address future research trajectories needed to better understand the signaling networks and cross talk in the signaling pathways of these molecules in cyanobacteria. Second messengers have significant potential to be adapted as technological tools and we highlight possible novel and practical applications based on our understanding of these molecules and the signaling networks that they control.

## 1. Introduction

**Figure 1:**
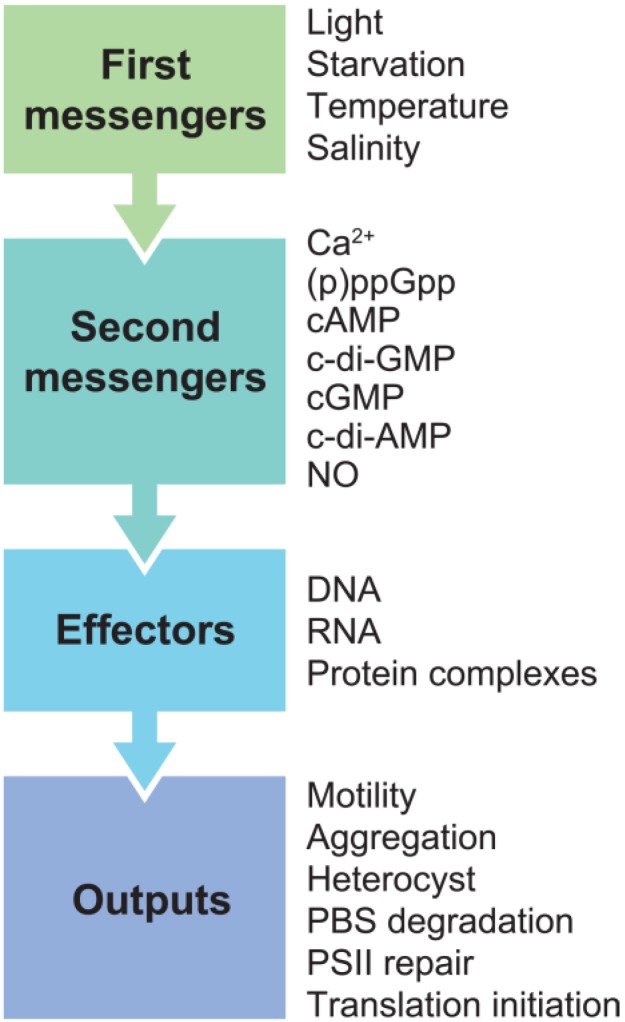
Specific second messengers are regulated by an external stimulus (*i.e.*, first messenger) and will bind specific effectors. In turn, the effector will initiate a signal cascade, which leads to organism-specific outputs (examples shown for cyanobacteria). This mechanism is common for all known second messengers.

Microorganisms need to cope with variations in the external environment and rely on signaling molecules to translate these changes into intracellular responses to mediate adaptation to the new condition. After cells sense an external stimulus (*i.e.*, first messenger), a second messenger will be synthesized or degraded to rapidly amplify the first messenger signal and initiate physiological changes. Proteins involved in the synthesis and degradation of second messengers are generally constitutively present in the cell to support rapid activation. First messenger-induced fluctuations of second messengers are propagated in cells through binding to DNA, RNA, or proteins/protein complexes. The ligand-effector complex will then trigger a signal cascade involving specific receptors, outputs and feedback processes. This signal cascade is common for all known second messengers (Figure 1). 

There is a rich variation of second messengers in prokaryotic organisms, from cyclic nucleotides to gases. In cyanobacteria, the most intensely studied second messengers are calcium (Ca^2+^), guanosine tetraphosphate or pentaphosphate (ppGpp or pppGpp; hereafter (p)ppGpp), and cyclic adenosine 3’,5’-monophosphate (cAMP). Lesser-studied second messengers in cyanobacteria are cyclic dimeric GMP (c-di-GMP), cyclic guanosine 3’,5’-monophosphate (cGMP) and nitric oxide (NO). By contrast, cyclic dimeric AMP (c-di-AMP) remains to be characterized in cyanobacteria. All of these second messengers are commonly studied in pathogenic bacteria. Calcium has been considered for its ability to influence cell structure and differentiation, motility, and gene expression in pathogenic bacteria [1]. The molecule (p)ppGpp is mainly studied in these bacteria for its involvement under cell starvation stress [2]. Cyclic AMP has been implicated in sugar metabolism [3], motility [4] and virulence [5] in pathogenic bacterial systems. Cyclic di-GMP impacts several processes in bacterial cells including transcription, RNA turnover, biofilm formation, protein synthesis, motility, and virulence [6]. NO serves as an important factor in host-pathogen interactions [7]. Finally, the most recently discovered nucleotide second messengers are c-di-AMP, which affects ion transport, membrane lipid homeostasis, DNA integrity [8], and cGMP, which has been implicated in the control of cyst formation [9].

Cyanobacteria are an ancient and distinct group of gram-negative photoautotrophic bacteria for which there have been limited investigations into the roles of most of these second messengers. Cyanobacteria are one of the most abundant photoautotrophic organisms in oceans [10] and are able to fix both carbon and nitrogen, thereby playing key roles in global carbon and nitrogen cycles. Elucidation of the processes controlling cyanobacterial adaption to aquatic habitats is critical for understanding their roles as primary producers controlling global carbon and nitrogen cycles. Cyanobacteria exhibit extraordinary diversity in terms of genome plasticity, morphological characteristics, ecological niches, and physiological properties [11,12,13]. These organisms generally rely on complex signal transduction systems, which reflect their diverse ecophysiological origins and abilities to colonize a wide range of habitats. Cyanobacteria, largely with the exception of marine *Prochlorococcus* and *Synechococcus* species, possess a much larger repertoire of two-component proteins compared to other bacteria [14], and they rely heavily on cyclic nucleotide signaling proteins [15,16,17].

In recent years, there has been a growing interest in utilizing cyanobacteria as systems for the production of valuable bioindustrial compounds from sugars to biofuels [18,19]. A number of natural physiological processes of cyanobacterial systems could be regulated to improve their use as bioproduction platforms. Sugar metabolism, motility, and biofilm production are just some examples of physiological processes under the control of second messengers. Regulatable control of these processes could lead to improvements in the efficiency of growing photosynthetic bacteria in partially or fully enclosed photobioreactors or other production platforms.

This review is designed to highlight the major advances in knowledge about the second messengers Ca^2+^, (p)ppGpp, cAMP, c-di-GMP, cGMP, c-di-AMP, and NO and their roles in cyanobacteria. Compared to other bacteria, the major contribution of these second messengers in cyanobacteria is to the regulation of key processes, such as nitrogen fixation, the perception of a variety of light qualities, photosynthesis-related processes, and gliding motility. This review aims to emphasize continuing areas of needed investigation for these signaling molecules and to address useful applications of knowledge about the signaling pathways to practical biotechnological interventions.

## 2. Second Messengers in Cyanobacteria

Studies on second messengers in cyanobacteria started ~40 years ago and have demonstrated that these molecules can influence several physiological processes. Genetic studies on the functional roles of second messengers have highlighted the involvement of these molecules in controlling physiological processes and biochemical studies and have described complex interactions between second messengers with DNA, RNA, proteins, and protein complexes.

### 2.1. Calcium, Ca^2+^

**Figure 2:**
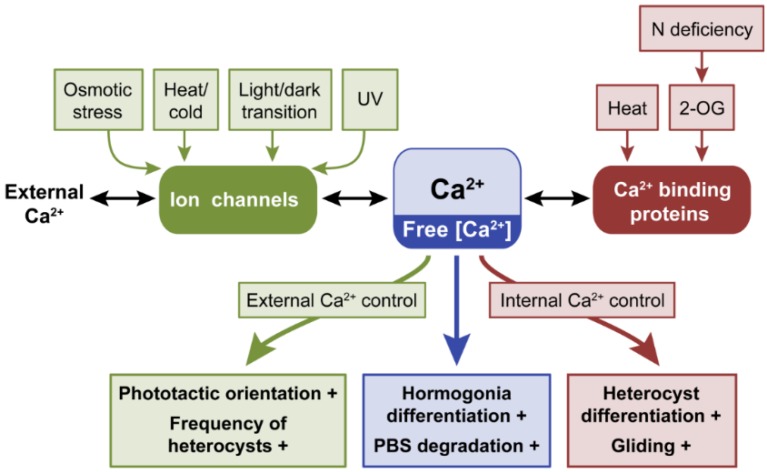
External signals controlling free intracellular Ca^2+^ levels and phenotypes or processes that are controlled by Ca^2+^ in cyanobacteria. Levels of free intracellular Ca^2+^ are regulated externally by influx(es) of extracellular Ca^2+^ through ion channels or internally by release of Ca^2+^ from Ca^2+^-binding proteins. Green arrow from free [Ca^2+^] indicates increased Ca^2+^ levels from external sources leads to noted phenotypes, whereas the red arrow indicates that increased Ca^2+^ levels from internal sources leads to the noted phenotypes. Blue arrow indicates that referenced studies did not demonstrate whether the noted phenotypes are under external or internal control. +, indicates process promoted by increased cellular Ca^2+^ levels; −, indicates process inhibited by increased intracellular Ca^2+^ levels.

One of the most intensely studied second messengers in cyanobacteria is the ion/element Ca^2+^. A role for Ca^2+^ as a second messenger in stimulus–response coupling has been correlated frequently with a variety of environmental stresses, such as heat and cold [20], oxygen stress [21], and osmotic stress [22]. It can impact a number of physiological responses, including motility, nitrogen fixation, and responses to stress [1]. Calcium must be tightly regulated to create a concentration gradient utilized by the cells to transfer information to downstream processes. Internal Ca^2+^ levels can be increased by an influx of Ca^2+^ present in the external medium, or by releasing intracellular stores of bound Ca^2+^ from Ca^2+^-binding proteins (Figure 2).

#### 2.1.1. Calcium Controls Motility

Calcium is involved in behavioral responses in prokaryotes, including directional motility in cyanobacteria [1,23,24,25]. The first reported study to investigate the effects of Ca^2+^ as a second messenger in cyanobacteria showed that gliding was attributed to an increase in cellular Ca^2+^ concentration [26]. A study of hormogonia, which are motile filaments of cells, confirmed that Ca^2+^ was critical to promote the differentiation of hormogonia and sustain motility [27]. In *Synechocystis sp*. PCC 6803 (hereafter *Synechocystis*), depletion of free Ca^2+^ resulted in diminished photoorientation and gliding speed [28]. Based on Ca^2+^ chelation and calcium ionophore studies, the authors concluded that phototactic orientation was likely caused by the uptake of extracellular Ca^2+^, whereas gliding motility was dependent on internal Ca^2+^ mobilization or release from Ca^2+^-binding proteins [28]. Using genetic and biochemical approaches, proteins containing Ca^2+^-binding domains were discovered [29,30,31]. A key step to understand gliding motility in cyanobacteria was to isolate surface fibrils. Fibrils in some cyanobacteria are composed of a single type of protein called oscillin, which contains multiple Ca^2+^-binding motifs [29]. *Phormidium uncinatum* filaments that did not accumulate oscillin were not able to glide [29]. Similar cell surface-associated glycoproteins function in this manner in other cyanobacteria [29]. Motility in *Synechococcus* sp. WH8102 depended on the protein SwmA, a homolog of oscillin that contains Ca^2+^-binding motifs. A mutant lacking *swmA* could still rotate but no longer exhibited swimming motility [30]. Calcium was also shown to promote swimming in *Synechococcus* strain WH8113 [31].

#### 2.1.2. The Role of Ca^2+^ in Heterocyst Differentiation

Calcium is required for the activity of the nitrogen-fixation enzyme nitrogenase *in vivo* and is purported to have a role in protecting nitrogenase from oxygen inactivation [32,33,34,35]. Moreover, the frequency of heterocysts found in a filament varies with different Ca^2+^ concentrations in the growth medium, which indicates a Ca^2+^-regulated mechanism for determining heterocyst abundance and placement [35]. These early experiments provided evidence that Ca^2+^ was involved in nitrogen fixation. 

The Ca^2+^-binding photoprotein aequorin can be used to measure intracellular levels of Ca^2+^ [36]. Determining the levels of free cytosolic Ca^2+^ is fundamental for establishing the role of Ca^2+^ as second messenger. Expression of an exogenous aequorin in *Anabaena* sp. PCC7120 (hereafter *Anabaena*) resulted in the detection of a distinct Ca^2+^ transient after nitrogen deprivation [37]. Alteration of the amplitude or duration of the Ca^2+^ transient using pharmacological treatments arrested heterocyst differentiation at an early stage [37]. Thus, proper regulation of the timing and amplitude of the transient promotes heterocyst differentiation [37]. Notably, an increase of Ca^2+^ in the cell after nitrogen deficiency originated from an intracellular source of Ca^2+^ [37]. 

Cyanobacteria can regulate Ca^2+^ homeostasis by using mechanosensitive ion channels and through protein-Ca^2+^ complexes that may serve in Ca^2+^ storage (Figure 2). The gene *hetR* in *Anabaena* encodes a calcium-stimulated protease essential for vegetative cells to differentiate into heterocysts [38]. In vegetative cells, intracellular free Ca^2+^ levels are ten times lower than in mature heterocysts [39]. Overexpression of *ccbP*, which encodes a Ca^2+^-binding protein that is localized in vegetative cells, suppressed heterocyst formation, whereas a *ccbP* mutant exhibited multiple contiguous heterocysts [39]. Thus, accumulation of CcbP in vegetative cells may contribute to sequestration of Ca^2+^ in these cells and its absence in heterocysts likely allows the accumulation of Ca^2+^ and associated induction of *hetR* and heterocyst differentiation [31].

Nitrogen deficiency is also an important signal mediated by Ca^2+^ in the unicellular, non-diazotrophic cyanobacterium *Synechococcus elongatus* PCC 7942 [40]. Cells under nitrogen deficiency produce 2-oxoglutarate (2-OG), an important biological compound involved in the carbon-nitrogen status signal. The anion 2-OG can trigger Ca^2+^ accumulation transiently in *S. elongatus* as an increase in 2-OG occurs in cells before observed changes in intracellular Ca^2+^ levels [40]. In response to nitrogen starvation, *S. elongatus* can degrade phycobilisomes to recyclenitrogen-rich amino acids through activating two transcriptional regulators, NtcA and NblR [41,42]. Under nitrogen deficiency, a transiently increased level of intracellular Ca^2+^ is NtcA dependent [40]. NtcA, which is a member of the cAMP-receptor transcriptional regulator protein family, may contribute to transient accumulation of 2-OG followed by Ca^2+^ in regulating the expression of NtcA-dependent genes involved in the process of phycobiliprotein degradation [40].

#### 2.1.3. Responses to Temperature Stress and Other Stresses Are Mediated by Ca^2+^

Calcium is also an important second messenger for cells to regulate responses to stresses, such as temperature shock, osmotic stress, and light-to-dark transitions [20,22,43]. The mechanosensitive ion channel protein MscL that is found in the plasma membrane of *Synechocystis* is involved in Ca^2+^ homeostasis regulation [44]. In *Anabaena*, heat shock at 44 °C resulted in an induction of intracellular Ca^2+^ levels that had a higher amplitude when Ca^2+^ was present in the external medium; similarly, a cold shock at 10 °C induced an increased magnitude of intracellular Ca^2+^ accumulation in cells [20]. The use of inhibitors or pharmacological agents indicated that the source of Ca^2+^ in the heat shock-induced elevation of cellular Ca^2+^ levels is both from Ca^2+^ in the external medium and internal stores, whereas the cold-shock induced elevation of Ca^2+^ levels results primarily from import of Ca^2+^ from the external medium [20].

Calcium is also induced by other stresses, including salinity, osmotic stress, light-to-dark transitions, and UV irradiation [22,43,45]. Intracellular levels of Ca^2+^ increased when *Anabaena* was exposed to salt or osmotic stresses. The source of increased intracellular Ca^2+^ levels was external as inhibiting calcium channels or the use of Ca^2+^-free medium eliminated this response [22]. Detecting changes in external light availability are critical for photosynthetic organisms, such as cyanobacteria. An increase of internal Ca^2+^ levels was observed in heterocysts in *Anabaena* when cells were exposed to UV irradiation [45]. A Ca^2+^ transient also occurred during a light-to-dark transition in *Anabaena* [43]. The observed elevation of intracellular Ca^2+^ levels was not associated with a specific photoreceptor, but more likely occurs in response to changes in the redox state of components of the photosynthetic electron transport chain [43]. Biochemical assays indicated that the source of the Ca^2+^ was external during the response to UV irradiation and light-to-dark transitions [43,45]. Together, the rapid Ca^2+^ transients that occur in cyanobacteria under temperature, osmotic, nitrogen and light stresses provide evidence that Ca^2+^ signaling is involved in early responses to these environmental stimuli. 

### 2.2. Guanosine-3’, 5’-(bis) Pyrophosphate, (p)ppGpp

**Figure 3:**
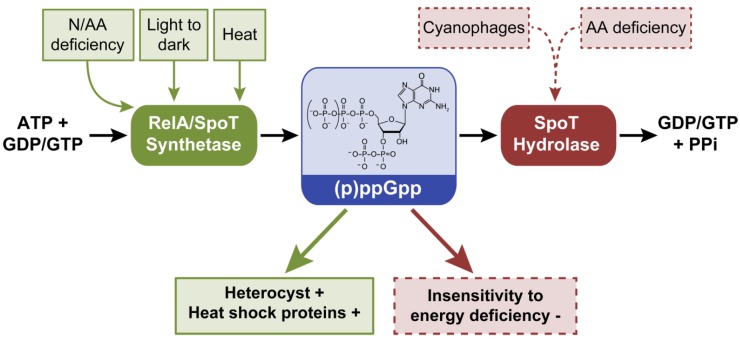
External factors controlling intracellular levels of (p)ppGpp and phenotypes or processes that are controlled by (p)ppGpp in cyanobacteria. (p)ppGpp is synthesized from GDP or GTP together with ATP by RelA or SpoT proteins and degraded to GDP or GTP and the by-product pyrophosphate (PPi) by SpoT. Dashed lines denote hypothetical or suggested role of cyanophages under nutrient-deficient growth conditions in controlling internal (p)ppGpp levels. Green arrow indicates that increased synthesis of (p)ppGpp leads to noted phenotypes, whereas red arrow indicates that degradation of (p)ppGpp leads to noted phenotypes. +, indicates process promoted by increased (p)ppGpp synthesis; −, indicates process inhibited by increased (p)ppGpp levels.

Guanosine-3’, 5’-(bis) pyrophosphate, (p)ppGpp, was the first second messenger characterized in cyanobacteria ~40 years ago. This molecule is involved in the stringent response, during which alterations in metabolism and gene expression occur due to limitation in the availability of amino acids or nutrition stress [39]. During the stringent response, resources are diverted away from growth towards amino acid synthesis to support survival until unfavorable conditions improve. During this process, ppGpp or pppGpp is synthesized from ATP and GTP/GDP by a (p)ppGpp synthetase, RelA [46], and can be degraded to GDP/GTP and pyrophosphate by a (p)ppGpp hydrolase, SpoT [46] (Figure 3). (p)ppGpp inhibits translation initiation to limit excessive protein synthesis during nutritional deficiencies [47]. Recently, more details of the regulation of (p)ppGpp and downstream processes emerged. In *E. coli* (p)ppGpp binds the β’ subunit of RNA polymerase and decreases the half-life of rRNA, resulting in decreased transcription [48]. (p)ppGpp also binds the β’ subunit of the plastid RNA polymerase in chloroplasts of plants [49], suggesting that (p)ppGpp likely binds the β’ subunit of the RNA polymerase in cyanobacteria, which are widely recognized as the progenitor of plastids [50].

The (p)ppGpp homeostasis enzymes RelA/SpoT (PF04607) are present as bifunctional gene homologs, sometimes referred to as RSH (Rel Spo homologs) genes [47], in each of the 83 finished genomes present in the Integrated Microbial Genomes (IMG) database [51] (genomes listed in Table S1) suggesting a widespread utilization of (p)ppGpp as a signaling molecule in cyanobacteria (Table 1). *Cyanobium gracile* PCC 6307, *Cyanothece* sp. BH68 ATCC 51142, and *Nodularia spumigena* CCY9414 possess two copies of the RSH genes annotated as RelA/SpoT (Table S2).

*Anabaena* possesses a single *relA*/*spoT* homolog (*all1549* or Ana-RSH) that regulates intracellular levels of (p)ppGpp in response to amino acid deprivation [52]. Ana-RSH was determined to be essential, as it could not be deleted from the genome [52]. Ana-RSH is maintained at a basal level in *Anabaena* under non-stressful conditions and appears to be regulated at the enzymatic rather than at a transcriptional level under amino acid deprivation [52]. As noted above, nitrogen deprivation leads to transient increases in (p)ppGpp levels in some cyanobacteria [53,54]; thus, the role of Ana-RSH during nitrogen deprivation and heterocyst development was explored. Although Ana-RSH was enriched in vegetative cells relative to heterocysts, the regulation of Ana-RSH or (p)ppGpp was not correlated with heterocyst formation and nitrogen fixation [52]. A more recent study, however, reported up-regulation of Ana-RSH under nitrogen deprivation and confirmed enrichment in vegetative cells [55]. These authors also were not able to delete Ana-RSH from the genome, but isolated a non-functional insertion mutant of Ana-RSH that exhibited reduced (p)ppGpp levels in response to amino acid starvation and failed to form heterocysts in nitrogen-limited conditions [55].

**Table 1:** Total number of genomes with specific pfam domains present in the finished genomes (n = 83) represented in the IMG database ^a^.

Second messenger	Pfam	Function	Number of genomes
**(p)ppGpp**	04607	(p)ppGpp synthesis and degradation	83
**cAMP or cGMP**	00211	Adenylate and guanylate cyclase	65
**c-di-GMP**	00990	Diguanylate cyclase	61
	00563	Diguanylate phosphodiesterase	60
**Nitric oxide**	00394	Nitrite reductase	27
	07731	Nitrite reductase	49
	07732	Nitrite reductase	47
	13442	Nitrite reductase	80
	00115	Nitric oxide reductase	83
**c-di-AMP**	02457	Diadenylyl cyclase	83

Note: ^a^ IMG, Integrated Microbial Genomes database [51].

#### 2.2.1. The Role of (p)ppGpp in Cyanobacterial Cells

The sources of carbon and energy are usually one and the same in heterotrophic organisms; however, in phototrophic organisms the source of energy, *i.e.*, light, differs from the source of carbon. Initial studies of (p)ppGpp in phototrophic organisms compared the effects of variations in light to nutrient starvation. The cyanobacterium *Synechococcus* sp. PCC 6301 (formerly *Anacystis nidulans*) responds to a reduction of ambient light, which is equivalent to an energy reduction, with reduced growth and an associated decrease in RNA synthesis and a transient increase in GTP levels. The levels of GTP decreased as (p)ppGpp began to accumulate [56]. Notably, amino acid deprivation induced similar responses in *Synechococcus* sp. PCC 6301 [57]. Nitrogen deprivation also transiently increased intracellular levels of (p)ppGpp in *Anabaena cylindrica* [53] and in *Synechococcus* sp. PCC 6301 [54]. Under nitrogen starvation in *Synechococcus* sp. PCC 6301, the regulation of (p)ppGpp is primarily due to (p)ppGpp synthesis rather than decreased degradation of (p)ppGpp [54]. In this organism, light-to-dark shifts and temperature stress led to accumulation of (p)ppGpp with an associated accumulation of heat shock proteins [58]. The effects of these two environmental factors could be separated, as ppGpp levels also increased when this species was exposed to 47 °C in the dark [58]. Thus, although the source of energy and carbon differ in cyanobacteria, a limitation of either results in a role of (p)ppGpp in transducing the environmental change.

#### 2.2.2. Cyanophage and (p)ppGpp

A fascinating aspect of (p)ppGpp regulation in cyanobacteria was observed when *Synechococcus* sp. PCC 6301 cells were infected with the AS-1 cyanophage (a cyanomyovirus). Cyanophage-infected *Synechococcus* sp. PCC 6301 cells failed to exhibit significantly increased intracellular (p)ppGpp levels under amino acid or energy deficiency as did uninfected cells, suggesting that infected cells did not perceive starvation [59]. One way for a cyanomyovirus to maintain low levels of (p)ppGpp is to express the protein MazG in the host; MazG is a protein found in all cyanomyovirus isolates [60]. In *E. coli*, MazG can hydrolyse (p)ppGpp [61], and if this is also true for MazG in cyanomyovirus-infected cyanobacteria, expression of MazG could allow the cyanomyoviruses to induce host cells to maintain basal levels of (p)ppGpp. Such a virus-induced response would support a normal cellular growth rate of the host, where the physiological state is expected to be optimal for production of progeny phage [62].

### 2.3. Cyclic Adenosine 3’,5’-Monophosphate, cAMP

**Figure 4:**
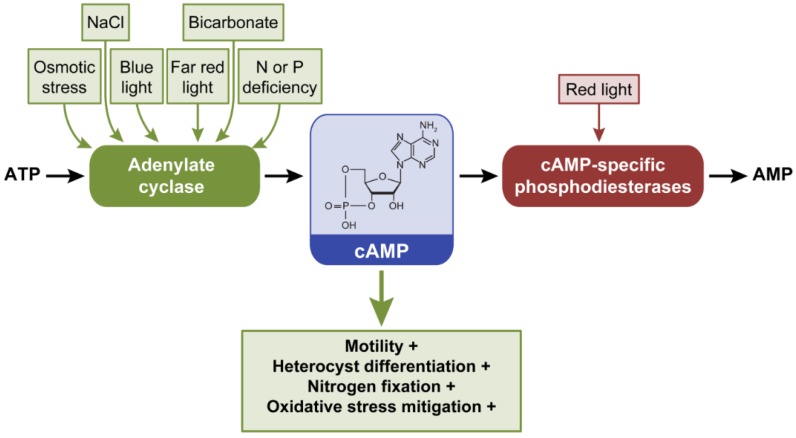
External factors controlling intracellular levels of cAMP and phenotypes or processes that are controlled by cAMP in cyanobacteria. Cyclic AMP is synthesized from ATP by adenylate cyclases (AC) and degraded to AMP by cAMP-specific phosphodiesterases. Green arrow indicates that increased synthesis of cAMP leads to noted phenotypes. +, indicates process promoted by increased cAMP levels.

Cyclic AMP is synthesized by adenylate cyclase (AC) (class III nucleotidyl cyclases family, Pfam: Pfam00211) proteins using ATP as a substrate and hydrolyzed to AMP by cAMP-specific phosphodiesterases (PDE) (Figure 4). Cyclic AMP is a widespread molecule in cyanobacteria. In an analysis of cyanobacterial genomes, only the picocyanobacteria *Prochlorococcus* and *Synechococcus* were reported to lack cAMP receptors, which can bind cAMP and serve as transcriptional regulators that impact diverse responses [17]. Species lacking cAMP receptors likely lost them during the course of evolution to adapt to new environments [17]. In an assessment of 83 finished cyanobacterial genomes present in the IMG database, AC genes homologous to the class III AC family (Pfam00211) were also found to be widespread (Table 1; Table S3), except in some strains that include mostly *Prochlorococcus* and *Synechococcus* strains (Table S4). 

#### 2.3.1. Cyclic AMP Regulates Motility Under Blue, Red, and Far Red Light

Cyclic AMP was initially recognized for its importance in controlling motility. In the filamentous cyanobacterium *Spirulina platensis*, external cAMP supplementation stimulated gliding motility and algal mat formation [63]. A *Synechocystis* mutant lacking the AC gene *cya1* exhibited lower intracellular levels of cAMP and did not exhibit movement on agar plates under standard white light growth conditions [64]. Further studies showed the blue light significantly and rapidly increased intracellular cAMP levels in *Synechocystis* compared to red or far red light [65]. Motility was enhanced by blue light in a fluence- and Cya1-dependent manner [65], indicating that a blue-light dependent photoreceptor was involved in the regulation of Cya1 during this process. 

Intracellular levels of cAMP are regulated by light in several cyanobacteria. Similar to *Synechocystis*, blue light serves as a crucial signal for cAMP regulation in *Microcoleus chthonoplastes* PCC 7420. A protein encoding a blue light-responsive LOV (light, oxygen, voltage) domain with an associated AC domain supported increased synthesis of cAMP *in vitro* upon exposure to blue light [66]. Whereas, blue light increases cAMP levels in *Synechocystis* and *M. chthonoplastes*, *Anabaena cylindrica* exhibits reversible red light-mediated reductions in cAMP levels and far-red light-induced increases in cAMP content [67]. Similarly, in *Anabaena*, red light decreased intracellular levels of cAMP, and far-red light levels increased cellular cAMP content [68]. Together, these findings suggest photoreceptor regulation of cAMP levels. Indeed, a mutant lacking a functional *aphC* gene, which encodes a phytochrome histidine kinase-like protein, did not exhibit FR-induced accumulation of cAMP [68]. In its photoregulation of intracellular cAMP levels, AphC purportedly phosphorylates and activates the AC CyaC, thereby inducing cAMP synthesis [68].

#### 2.3.2. cAMP-dependent Transcriptional Regulation of Motility

In *Synechocystis*, the cAMP-receptor protein SYCRP1 is required for motility and appears to impact pili biogenesis [69]. Non-motile *sycrp1* mutant cells showed a drastic change in the pili phenotype as the length and number of pili were extremely reduced. SYCRP1 exhibits cAMP-dependent binding to *E. coli* CRP consensus DNA binding sites [70,71]. Additional insights into the signaling pathway that controls motility emerged when it was demonstrated that several SYCRP1-regulated genes were under the control of Hfq, a homolog of a bacterial RNA-binding protein [72]. Notably, a mutant lacking *hfq* lost motility, which was correlated with the absence of pili on the cell surface. When SYCRP1 is activated it controls cell surface proteins CccS and CccP [73], both of which were also identified as targets of Hfq [69]. Similar to Δ*sycrp1* and Δ*hfq* mutants, a *Synechocystis* mutant lacking *cccS* showed an absence of thick pili [73].

#### 2.3.3. Cyclic AMP as Nutrient Deficiency Signal

Not only does cAMP control motility, but it is also an important signal for environmental nutrient deficiencies [74]. Nitrate and phosphate deficiencies resulted in transient increases in cAMP levels in *Anabaena flos-aquae* [74]. In *Anabaena variabilis*, an increased intracellular cAMP concentration was observed under nitrogen starvation that coincided with early heterocyst development [75]. Notably, cAMP can bind to AnCrpA, which in turn binds the 5’ upstream region of *nifB*, a nitrogen fixation-related gene, impacting its expression and that of other genes related to nitrogen fixation and heterocyst differentiation in the presence of nitrate [76]. Similar to SYCRP1, AnCrpA binds to a consensus CRP DNA sequence in a cAMP-dependent manner [77].

#### 2.3.4. The Role of cAMP under Other Stresses

An additional link between NaCl-induced cAMP signaling and heterocyst formation was also suggested [78]. NaCl, preferential to KCl or LiCl, transiently increased intracellular cAMP levels and induced expression of genes related to heterocyst formation [78].

cAMP levels transiently increase during rehydration following desiccation in *Anabaena* [79]. AC CyaC is important for this cellular response as a *cyaC* mutant is disrupted in cAMP accumulation and associated regulation of recovery from desiccation during rehydration [79]. This mutant exhibited impairments in oxygen evolution, increased ROS levels and increased respiration compared to wild-type cells [79]. Respiration in cyanobacteria can yield CO_2_, which equilibrates to bicarbonate in solution. Of note, bicarbonate causes a structural change in a cyanobacterial CyaC enzyme that has been shown to stimulate its AC activity and thereby result in increased cAMP synthesis [80]. A bicarbonate-stimulated induction of cAMP accumulation during rehydration could serve a protective role, as cAMP treatment of the *cyaC* mutant mitigated oxidative stress and growth impairments [79].

### 2.4. Cyclic Guanosine 3’,5’-Monophosphate, cGMP

**Figure 5:**
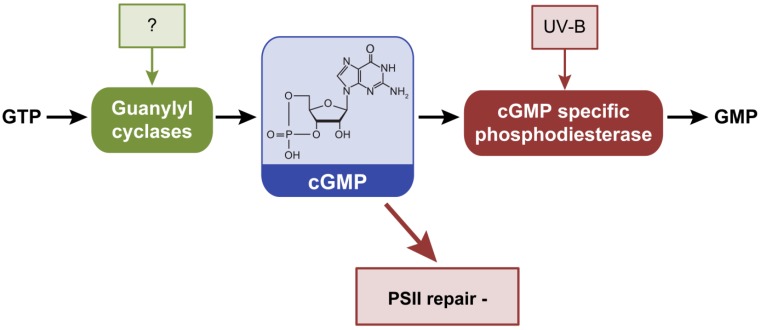
External factors controlling intracellular levels of cGMP and phenotypes or processes that are controlled by cGMP in cyanobacteria. Cyclic GMP is synthesized by guanylyl cyclases and degraded by cGMP-specific phosphodiesterases. −, indicates process inhibited by increased cGMP levels.

Cyclic GMP (cGMP) is a fairly recently confirmed second messenger in bacteria [81], although it has been well characterized in eukaryotic systems. Cyclic GMP is synthesized from GTP by guanylyl cyclases (GC), which are homologous to the class III adenylate cyclases. Class III AC and GC enzymes share similar catalytic domains and are thought to have evolved from a common ancestor [82]. Cyclic GMP is hydrolyzed to GMP by cGMP-specific phosphodiesterases (PDE) (Figure 5). Cyanobacteria contain higher levels of cGMP compared to other bacteria [83]. To date the only confirmed bacterial GC reported is the protein Cya2 from *Synechocystis* [84]. Mutational analysis of *cya2* indicated that the encoded protein contributes to intracellular cGMP levels, but does not impact cAMP levels [84]. Cya2 has higher specific activity for synthesizing cGMP from GTP than for the production of cAMP from ATP [85]. This cGMP specificity has been attributed to faster turnover of GTP than ATP by Cya2 rather than preferential affinity for GTP [85]. The only cyanobacterial phosphodiesterase (PDE) known to degrade cGMP is encoded by the gene *slr2100* in *Synechocystis* [86]. This cGMP-specific PDE is required for the adaptation of the cells to UV-B radiation. Intracellular cGMP levels decreased in wild-type after exposure to UV-B radiation, but not in the *slr2100* mutant. The *slr2100* mutant exhibited reduced transcripts of genes encoding components essential for PSII repair [86].

### 2.5. Cyclic Dimeric Guanosine 3’,5’-Monophosphate, c-di-GMP

**Figure 6:**
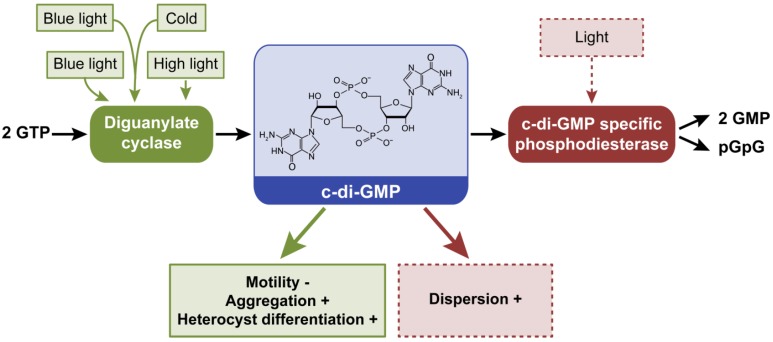
External factors controlling intracellular levels of c-di-GMP and phenotypes or processes that are controlled by c-di-GMP in cyanobacteria. Cyclic di-GMP is synthesized from two GTP by diguanylate cyclases and degraded to two GMP or pGpG by phosphodiesterases (PDE). Dashed lines denote hypothetical or suggested roles of light in activating PDEs and resulting phenotypes; these relationships between light absorption and c-di-GMP degradation are proposed as PDEs are often associated with photoreceptors in cyanobacteria [16] and this class of proteins induces motility and promotes dispersion in several pathogenic bacteria [6]. Green arrow indicates that increased c-di-GMP synthesis supports the noted phenotypes, whereas a red arrow indicates that increased degradation of c-di-GMP is associated with the noted phenotypes. +, indicates process promoted by altered c-di-GMP levels; −, indicates process inhibited by altered c-di-GMP levels.

Cyclic di-GMP is synthesized from two GTP molecules by diguanylate cylase (DGC) (Pfam 00990) activity, whereas c-di-GMP-specific PDE (Pfam00563 and Pfam01966) proteins degrade the molecule into pGpG or GMP (Figure 6). Proteins that can impact the synthesis or degradation of c-di-GMP and c-di-GMP-based signaling exist in a range of cyanobacterial species, yet are largely underexplored in these systems relative to their characterization in pathogenic bacteria [16]. Many cyanobacterial proteins contain light-responsive domains linked to domains that can impact c-di-GMP synthesis or degradation, suggesting that light is an important signal for altering c-di-GMP homeostasis and associated development, physiology and metabolism in cyanobacteria [16]. Among the species present in the CyanoBase database [87], the only species found to lack c-di-GMP signaling systems were *Prochlorococcus* and some strains of *Synechococcus* [16], similar to a report for cAMP [17]. DGC and PDE domain sequences (*i.e.*, Pfam00990 and Pfam00563) were used to identify conserved c-di-GMP domains in the 83 finished genomes in IMG (Table 1; Tables S5 and S6). These new results confirmed that only *Prochlorococcus* and some strains of *Synechococcus* lack c-di-GMP domains, with the exception of UCYN-A, an uncultured unicellular cyanobacterium associated with a eukaryotic cell [88] (Table S7). Similar to cAMP receptors [17], species adapted to stable habitats may have lost genes encoding c-di-GMP-modulating proteins [16].

To date, the protein encoded by gene *all2874* in *Anabaena*, the phytochrome-like protein Cph2 in *Synechocystis*, and the cyanobacteriochrome SesA in *Thermosynechococcus elongatus* are the only reported cyanobacterial proteins with a functional DGC activity characterized *in vitro* [89,90,91]. Mutation of the gene *all2874* resulted in decreased heterocyst differentiation and reduced vegetative cell size under relatively high light intensity [91]. Cph2 has three GAF domains, two DGCs and one PDE domain. GAF domains bind a light-absorbing chromophore in phytochrome family proteins [92,93]. Cph2 has been studied for its involvement in inhibiting phototaxis toward blue light in *Synechocystis* [89]. Although wild-type *Synechocystis* cells did not move toward blue light, mutants lacking Cph2 showed phototaxis toward the light source. Covalent binding of a tetrapyrrole to conserved cysteine residues has been shown for two of the GAF domains of Cph2, as has light-induced photoconversion [89,94]. The DGC SesA possesses one GAF domain that can sense green and blue light [90]. SesA is responsible for cell aggregation under blue light at relatively low temperature [90]. Although c-di-GMP levels were not measured *in vivo* in any of these studies, these results suggest that light can serve as a signal for regulating c-di-GMP levels in these three species.

Recently, we demonstrated that intracellular levels of c-di-GMP are regulated by light in some cyanobacteria *in vivo* [16]. Levels of c-di-GMP were higher under blue light than other qualities of light in *Synechocystis*, whereas c-di-GMP levels were lower under blue light and higher under white and red light in the chromatically-acclimating *Fremyella diplosiphon*. Intracellular c-di-GMP levels in *F. diplosiphon* were overall higher than those measured in *Synechocystis*. These data confirmed that light is an important first messenger for regulating this second messenger in cyanobacteria.

### 2.6. Nitric Oxide, NO

**Figure 7:**
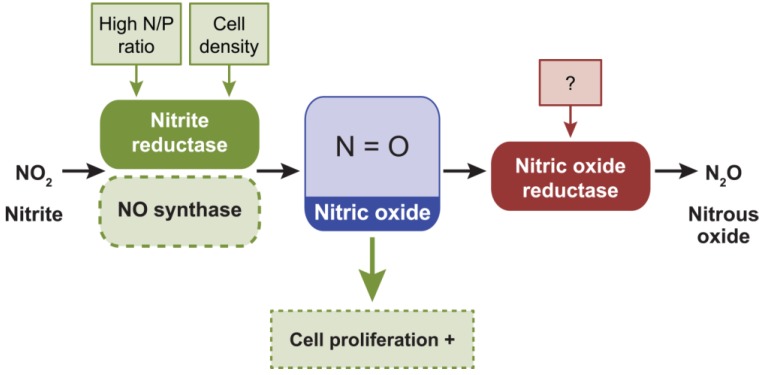
External factors controlling intracellular levels of nitric oxide (NO) and phenotypes or processes that are controlled by NO in cyanobacteria. NO is synthesized from nitrite (NO_2_) by nitrite reductase (and/or NO synthase) during denitrification and reduced to nitrous oxide (N_2_O) by nitric oxide reductase. Dashed lines denote hypothetical roles of NO synthase in controlling NO concentration. Green arrow pointing to the dashed green box indicates that cell proliferation could be induced (+) by high intracellular levels of NO [96].

Cyanobacteria are proposed to have contributed to the rise of NO in the atmosphere due to the production of ozone from the photolysis of O_2_, the latter of which is generated as a by-product of oxygenic photosynthesis [95]. NO is able to neutralize ozone, scavenge reactive oxygen species and mediate their potentially damaging effects [95]. NO is an intermediate of denitrification produced through reduction of nitrite by nitrite reductase by bacteria; NO can be reduced to nitrous oxide by nitric oxide reductase (Figure 7). Although exogenous NO can be a toxic gas, at low concentrations NO can be used as signaling molecule involved in the regulation of diverse biochemical and physiological processes.

A limited number of cyanobacteria have been shown to produce NO when grown in nitrate-containing media [97]. However, there are limited insights into the *in vivo* role(s) of NO as a second messenger in cyanobacteria. Increasing concentrations of NO were produced and released at higher cell densities in the cyanobacterium *Microcystis aeruginosa* [96]. Increasing the nitrogen-to-phosphorous ratio of the growth medium also supported higher levels of NO accumulation in this organism [96]. NO accumulation appears to be primarily due to the activity of nitrate reductase, rather than NO synthase in *Microcystis aeruginosa* [96]. In the 83 finished cyanobacterial genomes, nitrite reductase (Pfam00394, Pfam07731, Pfam07732, or Pfam13442) homologs are largely present in cyanobacteria (Table 1; Tables S8–S11). The nitric oxide reductase (Pfam00115) is present in all finished cyanobacterial genome (Table 1; Table S12).

### 2.7. New Second Messenger-Dependent Phenotypes in Cyanobacteria

**Figure 8:**
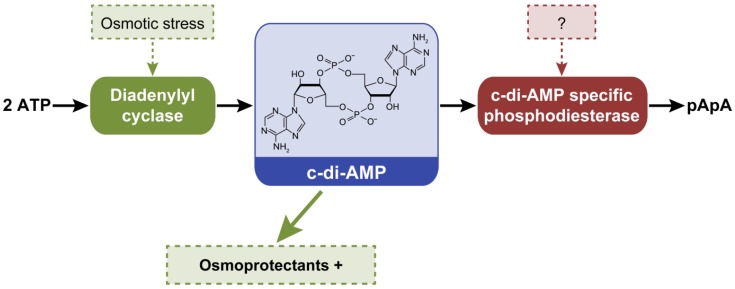
Suggested external factors controlling intracellular levels of c-di-AMP and phenotypes or processes that are proposed to be controlled by c-di-AMP in cyanobacteria. Cyclic di-AMP is synthesized from two ATP by diadenylyl cyclases and degraded to pApA by a putative phosphodiesterase. Green arrow pointing to the dashed green box indicates that the production of osmoprotectants could be induced (+) by increased c-di-AMP synthesis.

The DisA_N domain shows diadenylyl cyclase (DAC) activity and synthesizes c-di-AMP from two molecules of ATP; c-di-AMP is degraded to pApA by c-di-AMP specific PDE enzymes [8] (Figure 8). All cyanobacteria with a finished genome in IMG possess at least one DAC (PF02457) (Table 1). *Cyanothece* sp. PCC 7424, *Cyanothece* sp. PCC 7822, *Gloeobacter kilaueensis* JS1, *Gloeobacter violaceus* PCC 7421, and *Synechococcus* sp. PCC 7002 instead possess two DAC genes (Table S13). The presence of DAC in sequenced cyanobacterial genomes suggests an important role for c-di-AMP in these organisms. Surprisingly, DAC are orphan proteins; they have not been reported to be associated with other sensing domains. Cyclic di-AMP plays a central metabolic regulatory role in bacteria [98]. It has been suggested that c-di-AMP functions under osmotic stress in cyanobacteria based on assessment of regulons of riboswitches involved in binding c-di-AMP that include targets implicated in the transport and synthesis of osmoprotectants [99]. Cyclic di-AMP could also control the synthesis of c-di-GMP in some organisms, thereby potentially unveiling additional new roles of this widespread nucleotide [99].

Recently the hybrid cyclic dinucleotide c-AMP-GMP was discovered in *Vibrio cholerae* [100]. The protein DncV can produce c-AMP-GMP from ATP and GTP. To date, the physiological function of c-AMP-GMP remains unsubstantiated. Using the program BLAST, the best matching sequences from the 83 finished genomes present in the IMG database were the genes *Nos7107_0246* from *Nostoc* sp. PCC 7107 and *Glo7428_5202* from *Gloeocapsa* sp. PCC 7428. The statistically significant E-values for these genes were 9e^−11^ and 9e^−10^, respectively, with identities around 27% for both.

### 2.8. Cross Talk in Second Messenger Signaling

Interactions between signaling pathways creates a flexible signaling network that allows an organism to finely tune its responses to complex, and perhaps interacting, external stimuli. There are reported examples of regulatory cross talk between second messenger signaling pathways in cyanobacteria. For example, a partially purified adenylate cyclase was activated by Ca^2+^ in *Anabaena* sp. ATCC 29151 [101]. As cyanobacteria have been demonstrated to possess both cAMP and cGMP, a potential for cross talk between their regulatory networks emerges in such organisms. Indeed, in a cGMP PDE mutant that exhibits elevated cGMP levels, transcript levels were increased for AC gene *cya1*, guanylyl cyclase gene *cya2*, and a cAMP receptor protein-encoding gene [86]. These results provide evidence for crosstalk between some second messenger signaling pathways in cyanobacterial systems.

## 3. Challenges for the Future

### 3.1. The Complexity of Second Messenger Regulatory Networks

Second messenger homeostasis genes are widespread in the genomes of cyanobacteria and a single second messenger molecule can regulate several phenotypes. For instance, Ca^2+^ can control gliding motility, heterocyst differentiation, and degradation of phycobilisomes (Figure 2). (p)ppGpp regulates ribosomal RNA accumulation, heat shock proteins, and heterocyst differentiation (Figure 3). Cyclic AMP is involved in photoprotection, heterocyst differentiation, photoheterotrophic growth, and nitrogen and phosphorous uptake (Figure 4). In addition to a single second messenger controlling multiple phenotypes, multiple genes may encode proteins that regulate the synthesis or degradation of a single second messenger molecule. One extreme case is apparent with the c-di-GMP signaling system in bacteria. Among cyanobacteria possessing c-di-GMP proteins, there is an average of 20 enzymes that synthesize or degrade c-di-GMP for each species in which they are found [16]. There are several mechanisms by which c-di-GMP signaling specificity is achieved in systems exhibiting such complexity. One mechanism involves regulating the timing of accumulation of c-di-GMP relative to the presence of c-di-GMP receptors or effectors in cells. For instance, expression of c-di-GMP homeostasis enzymes could occur under environmental conditions that support accumulation of c-di-GMP specific targets. This has been demonstrated in *E. coli* for c-di-GMP in the control of biofilm formation during stationary phase [102]. Alternatively, in light of evidence that c-di-GMP enzymes are constitutively expressed in many species, a different mechanism could explain signaling specificity. In this instance, temporal or spatial sequestration of individual c-di-GMP components could be used to control signaling, in which case c-di-GMP molecules would target co-localized receptors. The use of c-di-GMP sensors has demonstrated distinct patterns of spatial localization of c-di-GMP in some bacterial cells [103]. Individual second messenger homeostasis enzymes also could distinctly effect second messenger pools and thereby control distinct phenotypes in the organism. Such signaling is referred to as high-specificity signaling and has been reported for distinct c-di-GMP synthesis enzymes in *Vibrio cholera* [104]. Also, by controlling the presence of receptors of a second messenger that have differences in binding affinities, the activation of distinct receptors could be achieved at different intracellular second messenger concentrations, thereby allowing specificity of control of distinct phenotypes [105]. Although compartmentalization of several second messengers has been well demonstrated in eukaryotic cells [106], signaling specificity in cyanobacterial systems has not been well investigated. Technologies already in use in pathogenic bacteria, such as fluorescence resonance energy transfer (FRET) [103] or single fluorescent protein-based indicators [107] could be used to monitor c-di-GMP or cAMP concentrations to permit visualization of asymmetrical distributions of these second messengers in cyanobacteria.

### 3.2. Second Messengers and Practical Application in Biotechnology or Therapeutics

Cyanobacteria have a realistic potential to generate fuels and high-value bioindustrial products using partially or fully enclosed bioreactors [108,109]. Biofuel, ethanol, isobutanol, alkanes, biodiesel, hydrogen, sugars, and medicinal products are just a few examples of compounds that cyanobacteria can produce (reviewed by [18,19]). Engineering cyanobacteria for efficient growth and harvesting is a priority to decrease the costs and reduce environmental trade-offs. Additional insights will provide a better understanding into the roles of second messengers in regulating specific aspects of cyanobacterial growth, including the impact of environmental factors and the ability to induce cellular floating or biofilm formation. Introducing exogenous enzymes that could regulate intracellular levels of second messengers under the control of inducible promoters could be an attractive tool for regulated growth of these organisms. For instance, cells could be induced to aggregate and deposit in the bottom or to float to the surface in partially or fully enclosed bioreactors by expressing exogenous enzymes to promote energy-efficient harvesting of biomass. In addition, the induction of biofilm formation could be applied in environments contaminated by positively charged heavy metal ions, as biofilms include exopolysaccharides, which have been considered useful for metal biosorption [110].

Second messengers that are not synthesized by eukaryotes have enormous engineering potential for use in mammalian cell therapeutics. Many potential applications are possible, including controlling the levels of second messengers through light-dependent mechanisms by associating second messenger homeostasis domains with a photoreceptor [111]. Proteins containing cAMP and cGMP synthesizing domains associated with photoreceptors have been engineered for potential optogenetic applications [111,112,113,114,115]; yet, as cAMP and cGMP are present in mammals these two second messengers may not be optimal for cell-based therapeutics. However, there may be potential for the development and use of light-activatable, nucleotide second messenger-degrading enzymes to regulate levels of these molecules *in vivo* for therapeutic purposes. Cyclic di-GMP or c-di-AMP are particularly interesting therapeutic targets as they are not present in mammalian cells and therefore are not expected to interfere with or alter native physiological processes [116]. Cyclic di-GMP and c-di-AMP can directly induce a STING protein-dependent response, which leads to the production of cytokines essential for the induction of an innate immunity response to bacterial infection [117]. Furthermore, cyanobacterial photoreceptor domains, such as the GAF domain can absorb a wide range of light wavelengths [118]; including wavelengths that represent regions of low light absorption in mammalian tissues [119]. Thus, there is significant potential for the development of optogentic tools based on the large number of putative light-responsive c-di-GMP homeostasis proteins found in cyanobacteria [16].

## 4. Concluding Remarks

To date, second messengers have been shown to play key roles in controlling fundamental and perhaps underappreciated aspects of photosynthetic-related processes. Heterocyst differentiation is regulated by a complex signaling network involving cAMP, Ca^2+^, and (p)ppGpp. Phototaxis is regulated by both cAMP and c-di-GMP, whereas UV photoprotective mechanisms are regulated by cAMP and cGMP. Second messengers in cyanobacteria also function in pathways mediating cellular responses to oxidative stress, nutrient imbalances, and temperature variations in the environment. In the future, new studies are anticipated to shed light on additional phenotypes that are under control of second messenger molecules. Elucidation of the roles of uncharacterized second messengers, including c-di-AMP, is anticipated to provide additional insights into the complex biological networks and physiological responses regulated by second messengers. Such additional knowledge gained about second messenger signaling pathways may support the development of new tools for biotechnological, optogenic, and therapeutic applications.

## Supplementary Materials

Supplementary materials can be accessed at: http://www.mdpi.com/2075-1729/4/4/745/S1.
